# Using EASE to Identify Characteristics That Distinguish Traditional Versus Person Centered Living Areas

**DOI:** 10.1093/geroni/igaf122.624

**Published:** 2025-12-31

**Authors:** Maggie Calkins, Adam Davey, Migette Kaup, Robert Wrublowsky

**Affiliations:** IDEAS Institute, Cleveland Heights, Ohio, United States; University of Delaware, Newark, Delaware, United States; Kansas State University, Manhattan, Kansas, United States; RAW Consulting, Winnipeg, Manitoba, Canada

## Abstract

The Environmental Audit Screening Evaluation tool (EASE) is a unique assessment tool which was administered in 228 living areas across US and Canada. One-way analysis of variance was used to estimate eta-squared values as indicators of effect sizes between settings that were identified as being either traditional or homelike. Elimination of items with eta-square values 1.0 or less resulted in 39 items that maximally distinguished between different types of settings. These 39 items were subjected to parallel analysis which generated a 4-component solution that provided high loadings with few cross-loadings. Specifically, this subset of EASE items appeared to assess: 1) residential and familiar character (14 items) (e.g., kitchen design appears residential in style, and required institutional features (such as exhaust vents) are disguised) 2) orientation and functional supports (15 items) (e.g., doors to dangerous storage largely out of sight) 3) human scale and intuitive layout (8 items) (e.g., bedrooms close to social spaces) and 4) autonomy in daily living (8 items) (e.g., showers allow privacy and independence). The value and use of this subset of items as a tool in multi-site research will be discussed.

